# Do Laying Hens with Keel Bone Fractures Experience Pain?

**DOI:** 10.1371/journal.pone.0042420

**Published:** 2012-08-22

**Authors:** Mohammed A. F. Nasr, Christine J. Nicol, Joanna C. Murrell

**Affiliations:** 1 School of Veterinary Sciences, University of Bristol, Bristol, United Kingdom; 2 Department of Animal Wealth Development, Faculty of Veterinary Medicine, Zagazig University, Sharkia, Egypt; University of Kentucky Medical Center, United States of America

## Abstract

The European ban on battery cages has forced a change towards the use of non-cage or furnished cage systems, but unexpectedly this has been associated with an increased prevalence of keel bone fractures in laying hens. Bone fractures are acutely painful in mammals, but the effect of fractures on bird welfare is unclear. We recently reported that keel bone fractures have an effect on bird mobility. One possible explanation for this is that flying becomes mechanically impaired. However it is also possible that if birds have a capacity to feel pain, then ongoing pain resulting from the fracture could contribute to decreased mobility. The aim was to provide proof of concept that administration of appropriate analgesic drugs improves mobility in birds with keel fracture; thereby contributing to the debate about the capacity of birds to experience pain and whether fractures are associated with pain in laying hens. In hens with keel fractures, butorphanol decreased the latency to land from perches compared with latencies recorded for these hens following saline (mean (SEM) landing time (seconds) birds with keel fractures treated with butorphanol and saline from the 50, 100 and 150 cm perch heights respectively 1.7 (0.3), 2.2 (0.3), p = 0.05, 50 cm; 12.5 (6.6), 16.9 (6.7), p = 0.03, 100 cm; 20.6 (7.4), 26.3 (7.6), p = 0.02 150 cm). Mobility indices were largely unchanged in birds without keel fractures following butorphanol. Critically, butorphanol can be considered analgesic in our study because it improved the ability of birds to perform a complex behaviour that requires both motivation and higher cognitive processing. This is the first study to provide a solid evidential base that birds with keel fractures experience pain, a finding that has significant implications for the welfare of laying hens that are housed in non-cage or furnished caged systems.

## Introduction

A European ban on conventional (‘battery’) cages for laying hens came into effect in January 2012, which over the last 10 years has forced a change towards the use of non-cage or furnished cage systems. These systems allow birds greater freedom of movement compared with conventional cages, however an unexpected consequence of the change in housing type has been an increase in the prevalence of keel fractures in laying hens [1]. A recent study [2] reported a very wide range in prevalence of keel fractures across different housing systems, from 36% in furnished cages to 86% in free-range systems where aerial perches were suspended in the indoor house. It is not easy for a farmer to identify hens with fractures and hen survival rate seems high. Thus the effect of both recent and healed fractures on bird welfare is unclear.

Bone fractures are reported to be acutely painful in humans [3]. The immediate pain is mainly attributed to activation of mechanosensitive nociceptors in the periosteum of the affected bone due to fracture displacement [4]. Influx of inflammatory cells to the fracture site follows rapidly, resulting in the release of inflammatory cytokines which further stimulate nociceptors. Tissue damage and continued activation of nociceptors can trigger both peripheral and central sensitization, manifest as hyperalgesia, allodynia and spontaneous pain [5]. Mechanical stimulation of the periosteum (e.g elicited by movement) is a potent trigger of pain [6]. Chronic pain may persist after the fracture has healed, particularly if there has been injury to sensory nerves [7], [8]. Bone fractures cause severe acute pain in other mammals [9]–[11] although the incidence of chronic pain arising from healed fractures in mammals other than humans is unknown.

The sensory innervation of the keel bone in birds has not been studied, but similarities in bone physiology and fracture healing in mammals and birds strongly suggests that the keel bone will be densely innervated by sensory afferent fibers. The keel bone is vulnerable to movement caused by flight or perching [12] so that these normal behaviours are likely to cause disruption of any acute keel bone fracture and generate nociceptive activity.

We recently reported that birds with keel bone fractures take longer to reach a food reward in a runway test and took approximately 4 times as long to fly down from a perch to obtain a food reward than hens with no keel bone fractures [13]. Also, hens with keel bone fractures of mild severity (severity score 1, [12]) took a shorter time to finish the tests successfully than hens with severe fractures (severity score 2, [12]). Thus keel fractures have a distinct effect on mobility, although the cause of this decreased mobility remains to be investigated. One possible explanation is that walking and flying become mechanically impaired by an anatomical deformity of the keel bone and damage to the pectoralis major and supracoriacoideus muscles that insert on it, causing subsequent muscle degeneration or disuse atrophy. The keels of birds with healed keel fractures were colder than those of birds with normal keels, assessed using infra-red thermography [13], supporting this hypothesis. However it is also possible that if birds have a capacity to feel pain, then ongoing pain resulting from fracture of the keel bone could contribute to decreased mobility.

Pain may be inferred in animals by observing changes in their behaviour, either the cessation of abnormal behaviours or the resumption of normal behaviours when drugs that are known to be analgesic in man are administered [14]–[16]. The aim of this study was to provide proof of concept that the administration of appropriate analgesic drugs might reverse the impairment of mobility observed in birds with keel fracture. Such a result would contribute to the general debate about the capacity of birds to experience pain, as well as to the specific debate about whether fractures are associated with chronic pain in laying hens. Two different classes of opioids, a kappa agonist butorphanol and the full mu agonist morphine, were administered to two groups of birds, one with and one without keel fractures and their mobility was assessed.

## Results

The mean (SEM) bodyweights of birds with and without keel bone fractures on arrival at the experimental site were 1.79 (0.02) and 1.82 (0.03) kg respectively, this difference was not statistically significant (p>0.05). For all hens, keel bone fracture status was reclassified post mortem following keel bone dissection. We found 23 birds without keel bone fracture and 35 birds with a keel bone fracture.

### Morphine

Morphine increased the time to land in all the birds, whether they had fractures or not, from the 100 and 150 cm perch heights, but not from 50 cm, compared to saline. Overall, birds treated with morphine took approximately 3 times longer to fly down from 100 cm perches (mean (SEM) landing time (sec) 32.0 (10.1) morphine, 11.6 (5.2) saline, F = 10.08, p<0.001) and 1.5 times longer to fly down from 150 cm perches (mean (SEM) landing time (sec) 49.5 (11.8) morphine, 34.0 (9.8) saline, F = 3.89, p = 0.05). In addition, birds with keel fractures took approximately 6 times longer to fly down from the 100 cm perches (mean (SEM) landing time (sec) 6.6 (11.4) birds without keel fractures, 37.0 (9.3) birds with keel fractures, F = 4.25, p = 0.04) and 8 times longer to fly down from the 150 cm perches, (mean (SEM) landing time (sec) 9.9 (15.7) birds without keel fractures, 73.6 (12.8) birds with keel fractures, F = 9.88, p<0.001) than hens without fractures, although there was no statistical difference with the low, 50 cm perch. No interaction between the effects of morphine on landing time and the presence of a fracture was found for any perch height. There was no significant effect of order of drug treatment (whether morphine or saline was administered first) on landing time (p>0.05). Morphine caused the birds to become sedated, and when not stimulated, the birds adopted sleep like postures.

### Butorphanol

**Figure 1:**
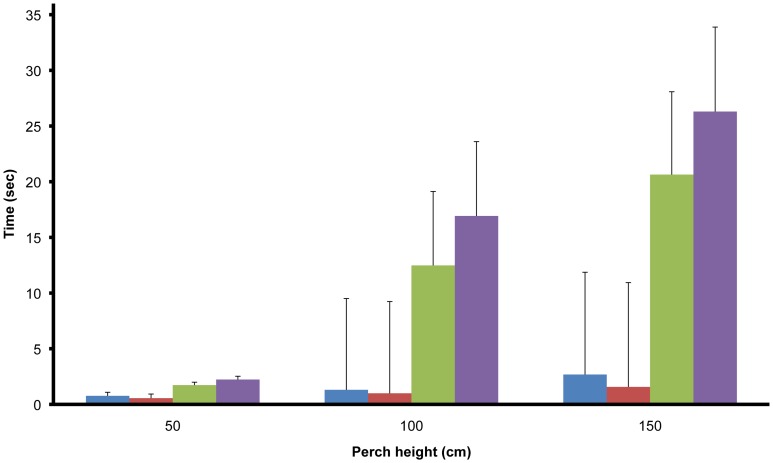
Latency to land from different perch heights after saline and butorphanol treatment. Mean (SEM) latency to land (seconds) from three different perch heights (50, 100, 150 cm) in birds with (n = 35) and without (n = 23) keel bone fractures following treatment saline or butophanol, 2 mg/kg injected subcutaneously in the dorsal neck. Birds without keel fractures are indicated in red (following saline treatment) or blue (following butorphanol treatment). Birds with keel fractures are indicated in purple (following saline treatment) or green (following butorphanol treatment).

There was a significant interaction between presence or absence of a keel fracture and the effects of butorphanol at all perch heights (50 cm: F = 3.71 p = 0.05; 100 cm: F = 4.72, p = 0.03; 150 cm F = 5.53, p = 0.02; df 1,54 in all cases). In hens with keel fractures, butorphanol decreased the latency to land from the different perch heights by approximately 20% compared with the latencies recorded for these hens following saline (Figure 1). However even with butorphanol treatment the latency to land remained significantly greater for hens with fractures than hens without. Butorphanol administration to hens without keel fractures either marginally increased (50 cm and 150 cm perch heights) or decreased (100 cm perch height) latency to land, but the magnitude of these effects were minimal and unlikely to be of any biological significance compared to the decrease in latency shown by hens with keel fractures. There was no significant effect of order of drug treatment (saline or butorphanol first) on landing time (p>0.05). Butorphanol did not cause any obvious effects on spontaneous behaviour and no sedation was apparent.

Post mortem of birds at the end of the study confirmed that all birds were correctly attributed to group (fracture or no fracture), and no birds classified without fractures at the start of the study developed a fracture during the course of the experiment. The average keel bone fracture severity score was 1.94.

## Discussion

Due to the unique qualities of pain as both a sensory and emotional experience, the ability of animals to experience pain, as opposed to nociception, cannot be ascertained without doubt. However there is some acceptance within the scientific community that non-human mammals might be capable of experiencing pain [17]. This is attributed in part to the similarities in the central nervous system between human and non-human mammals, although these similarities in brain structure alone do not provide sufficient evidence for the capacity of non-human mammals to experience pain. In humans, functional imaging and electroencephalographic studies consistently show activation of defined cerebral cortical structures during the administration of stimuli reported to be painful by the experimental subject, for example the primary and secondary somatosensory cortices, amygdala, anterior cingulated cortex and insula [18]. However these structures are also active during processing of non-painful, but salient, stimuli, such as auditory or visual cues [19], therefore the specific neural mechanisms leading to the experience of pain remain to be elucidated. Despite this uncertainty, it is widely acknowledged that an intact cerebral cortex is required for pain perception in mammals [20].

In contrast, although birds possess the neuroanatomical pathways necessary for nociception [21]–[23] there are opposing views as to the capacity of birds to experience pain [24]–[26]. An intact pallium, the functional equivalent of the cerebral cortex of mammals [27] may be a necessary prerequisite for birds to have the capacity to experience pain. However, the existence of an intact pallium alone does not provide sufficient evidence of pain capacity.

Further evidence can be obtained by examining the effect of administration of drugs with known antinociceptive properties. When arthritis is experimentally induced (by injection of sodium urate into the legs of chickens), behaviours such as limb guarding are increased, whilst normal locomotry and feeding behaviours are reduced [28]–[31]. Administration of intra-articular bupivicaine [28] systemic steroids [30] or non steroidal anti-inflammatory drugs (NSAIDs) [31] restored the frequency of some of these behaviours to control values. In other bird species, butorphanol has also proved antinociceptive in experimentally induced arthritis [32]–[34]. Collectively these results suggest that birds have the capacity to experience pain, although caution is required. Gentle et al. (1997) [24] found that decerebrate birds showed the same repertoire of “pain” related behaviours as intact birds. This finding strongly calls into question the assumption that behaviours exhibited by birds with arthritis can be attributed to the experience of pain, rather than nociception. In addition, such pain models are limited by their inability to produce key pathophysiological processes that underlie the development of chronic pain states [35].

In contrast to previous studies, the design of our experiment allows us to draw firmer conclusions about the capacity for birds to experience pain. We studied the effect of administration of drugs known to be analgesic in man, on a complex, spontaneous behaviour following bone fracture in laying hens. There has been no previous work to establish whether fractures are painful in chickens, although related studies have shown that some analgesic drugs can reverse some of the behavioural signs of clinical lameness in broiler chickens [36]. However, results are inconsistent and difficult to interpret [37].

Our experiment revealed that butorphanol, a synthetic kappa (κ) agonist and mu (µ) antagonist [38] substantially increased mobility indices in hens with keel fractures compared with saline treatment. In contrast, mobility indices were largely unchanged in birds without keel fractures following butorphanol administration. Critically, the effect of butorphanol can be considered analgesic in our study because it improved the ability of birds to fly from a perch to the ground; a complex, spontaneous behaviour that requires both motivation and higher cognitive processing. Thus we tentatively conclude that birds with keel fractures experience pain. The discriminatory effect of butorphanol on bird mobility, dependent on fracture status, also supports the contention that butorphanol improved mobility through provision of analgesia in birds with keel bone fractures. The design of the present experiment does not allow us to distinguish between whether butorphanol had an analgesic or an antihyperalgesic effect in this model. Bone fractures cause hyperalgesia in man [3], therefore it is reasonable to postulate that birds with keel bone fractures are also hyperalgesic. Opioids are considered to have both analgesic and antihyperalgesic effects, although the antihyperalgesic effects are considered to be of less profound, particularly for Mu agonist opioid drugs. The relative potency of an analgesic: antihyperalgesic effect of butorphanol in birds is unknown, but an antihyperalgesic effect of butorphanol may contribute to an improved treatment of pain states dominated by central sensitization such as pain resulting from bone fractures.

In mammals, kappa agonists produce dysphoria and a feeling of unpleasantness, however the effects of kappa agonists on mood and behavior in birds are unknown. It is also possible that butorphanol improved the mobility of the hens with keel bone fractures indirectly through an effect on behavior, rather than via a direct analgesic effect mediated through kappa opioid receptors in areas of the central nervous system associated with nociceptive processing and pain. Studies investigating the effects of other classes of analgesic drugs in this pain model, for example, non steroidal anti-inflammatory drugs, would help elucidate the underlying mechanisms associated with the increased latency to land time in birds with keel fractures.

Birds have a higher density of κ opioid receptors in the central nervous system compared with mammals [39] which may explain the efficacy of this class of opioid drug in birds [32], [40]. The antinociceptive effects of butorphanol have not been previously investigated in chickens but the dose of butorphanol administered in the present study is similar to doses that have proved antinociceptive in other bird species [32]–[34]. It was not the aim of the present investigation to determine the optimal dose of butorphanol for analgesia in birds with keel fractures. It is possible that the mobility of hens with keel fractures would have been further increased by a different dose of butorphanol, depending on the relative balance between the potential for increased analgesic efficacy with a higher dose versus the potential for increased sedation. The pharmacokinetics of 2 mg/kg butorphanol IV have recently been investigated in broiler birds [41]. These data suggested that the onset of analgesia after butorphanol administration SC would be in the order of 30 minutes and that the duration of analgesia would be short, hence the timing of assessments after butorphanol administration in the present investigation.

Morphine is analgesic in mammals and is widely used for provision of analgesia for acute pain states [42], [43]. In chickens the antinociceptive effects of morphine, administered at varying doses, have been studied in acute models delivering noxious mechanical or thermal stimuli and both antinociception [44]–[46] and pronociception [47], [48] has been reported. Morphine increased landing time in all the birds in the present study, an effect we attribute to sedation. Sleep-like behaviour and motor inco-ordination has been reported previously in birds following morphine [49], [50]. The dose of morphine was chosen based on a recent pharmacokinetic study of morphine in broiler chickens [51] that reported only minor sedation following intravenous administration of 2 mg/kg morphine. The profound sedation exhibited by our birds was, therefore, unexpected.

In our experiment all birds were treated with morphine or saline first followed by butorphanol or saline eight days later. The rapid clearance of morphine in birds [51] suggests that there would have been no residual effects of morphine during the butorphanol phase of the study, confirmed by return of normal behaviour, without concurrent sedation, a few hours after morphine administration. Some healing of fractures is likely to have occurred over the eight day period, and it is possible that birds with fractures developed new additional fractures during the experimental period [12]. However fracture healing should have reduced the differential effects of butorphanol on the mobility of birds with and without keel fractures. Despite this, a significant interaction was still identified. No bird without fractures at the start of the study developed a fracture during the course of the experiment, but it was impossible to determine at post-mortem whether birds with fractures at the outset developed new fractures. By the butorphanol phase of the study, birds were more familiar with the test procedure, evidenced by their shorter latencies to land from all three perch heights after saline injection in the butorphanol phase than after saline treatment in the morphine phase. Although the data for morphine and butorphanol were analysed independently of each other we cannot discount the possibility that prior exposure to morphine confounded the effects of butorphanol administration to birds with keel fractures, influencing our experimental results. The investigator was unaware of the fracture status of individual birds at the time of carrying out the landing test, but was not blinded to the treatment received by each bird (opioid or saline). Blinding to drug treatment would have been difficult because of the obvious behavioural effects caused by morphine administration to the hens. However because latency to land is an objective measure of bird mobility it is unlikely that the absence of blinding biased the data generated in this experiment.

## Conclusions

This is the first study to provide a solid evidential base that birds with keel fractures experience pain, a finding that has significant implications for the welfare of laying hens that are housed in non-cage or furnished caged systems. It is not appropriate or achievable to provide analgesia to birds with keel fractures in commercial housing systems. Rather the results of this study provide a compelling argument to prevent the development of keel fractures via genetic selection for more resilient bones, modified feeding and production practices and/or improved housing system design.

## Methods

### Ethics statement

The study was carried out under the Animals (Scientific Procedures) Act (PPL 30/2865). For experiments carried out under Home Office License local ethical approval by the institution is not required.

### Birds and management

Sixty two Lohman Brown strain of laying hens (aged 35 weeks) were obtained from a commercial farm; they were all assessed by palpation of the keel [1] to detect those with suspected healed keel fractures. Twenty-eight hens were selected with keel fractures, and 34 without evidence of fractures were selected as controls. They were randomly divided into two groups, each containing a similar number of hens with or without fractures. Hens were weighed on arrival at the experimental site. After allowing three days acclimatisation at the experimental site, the experiment was started for group 1 (31 hens), while group 2 did not undergo testing until 10 days after arrival, the experimental protocol was identical for both groups of hens. Each group was housed in a floor pen (3 m×3.5 m).

### Study protocol

We subjected the birds to a landing test [13] to investigate whether the test drugs altered the time the hens took to fly down from different perch heights to the ground to access a food reward. All the hens were initially trained by placing them on a low perch (20 cm) and allowing them to see a meal-worm. Hens were considered trained when they jumped from this low perch in less than 10 s and ate the food reward. Four birds (2 from each group) could not be trained to complete the landing test.

Trained hens in each group were randomly allocated to receive treatment with either morphine or saline first. Hens that received the morphine treatment were injected subcutaneously in the dorsal neck with morphine 2 mg/kg (morphine sulphate, 10 mg/ml solution) while the remaining hens were injected with an equivalent volume of saline. The hens were left for 30 minutes before starting the landing test. Each landing test comprised three trials with the perch set at 50 cm, 100 cm and finally 150 cm from the ground. After a 1 day rest period the landing test repeated following a second treatment with either morphine or saline. Birds that received morphine as the first treatment received saline as the second treatment, while birds that received saline first were treated with morphine. This sequence was then repeated such that each hen completed the landing test twice with morphine and twice with saline in a cross-over design. A rest interval of 8 days after final morphine administration was allowed to ensure that the drug was completely metabolised and cleared from their system.

Following the rest interval, the test procedure was repeated to examine the effect of butorphanol (2 mg/kg (Torbugesic 1% W/V solution for injection) injected subcutaneously in the dorsal neck) or saline administration in a second cross over design experiment. As before, the injected hens were left for 30 minutes before starting the test. Each hen completed the landing test twice with butorphanol and twice with saline, with a 1 day rest period allowed to elapse between administration of either butorphanol or saline.

The mean time taken to land was calculated for each drug and for saline for each bird, prior to statistical analysis.

Thirty eight days (group 1) and 48 days (group 2) after arrival birds were killed humanely using a Schedule 1 method and each was examined post-mortem to determine the extent and nature of any keel bone pathology. If keel fractures were detected their severity was rated as either mild (severity score 1) or severe (severity score 2) [12].

### Statistical analysis

Statistical analysis on the mean time taken to land was carried out using PASW Statistics (SPSS version 18.0 for Windows). For each perch height, a General Linear Model Repeated Measures design was used. We examined the effect of two between-subjects factors (i) the keel fracture status of the bird (fracture/no fracture) and (ii) the order of drug presentation (butorphanol tested before or after saline). The effect of butorphanol or saline administration was examined as the repeated measure for each bird. Values were considered significant at p≤0.05.
